# P-69 - MODELLING VACCINATION STRATEGIES AGAINST GONORRHOEA IN THE BRITISH POPULATION

**DOI:** 10.1093/pubmed/fdag046.092

**Published:** 2026-07-09

**Authors:** Mr Soeren Metelmann, Dr Feng Xu, Dr Segun Oke, Ms Suzy Sun, Dr Luana Lenzi, Dr Valerie Decraene, Dr Alex Thompson, Dr Ray Borrow, Dr Roberto Vivancos, Dr Lorenzo Pellis, Dr Ian Hall

**Affiliations:** UK Health Security Agency; University of Manchester; University of Manchester; UK Health Security Agency; University of Manchester; UK Health Security Agency; University of Manchester; UK Health Security Agency; UK Health Security Agency; University of Manchester; University of Manchester

## Abstract

**Background:**

Gonorrhoea is a sexually transmitted infection caused by the bacterium Neisseria gonorrhoeae. With case numbers resurging in many countries and the emergence of drug-resistant strains it is a significant public health concern. Development of a vaccine against this infection proofed difficult for decades until observations of a meningococcal B vaccine being partially effective against gonorrhoea has given new hope. In 2023, JCVI even recommended the use of MenB vaccines for the risk group of gay, bisexual, and other men who have sex with men (GBMSM). For other population groups though, the usefulness of the vaccine remains less clear.

**Objectives:**

Our main aim was to develop a model that can replicate and project gonococcal infections in the whole of the British population and simulate different vaccination strategies with MenB vaccines. In a second step, we then looked at the cost effectiveness of each strategy.

**Methods:**

We use a deterministic mathematical model to describe gonococcal infections in four sexually active groups: women, heterosexual men, and GBMSM with a low and with a high number of sexual partners. For the health economic model, we perform a basic cost-benefit analysis comparing the cost of the vaccination programmes against the economic burden of disease and the quality adjusted life years lost.

**Results:**

Our model Modelling results suggest that a vaccine that is even partially protective against gonorrhoea, delivered through an effective targeting strategy, could indeed have a significant impact on reducing the incidence of the infection. A treatment centred strategy is cost effective across all scenarios and sensitivity tested.

**Conclusions:**

We show that vaccination at screening would be most effective in terms of disease control. The greatest benefits are observed when vaccine is targeted at GBMSM groups because of the eventual reduction in female cases.

